# pH-Induced Folding of the Caspase-Cleaved Par-4 Tumor Suppressor: Evidence of Structure Outside of the Coiled Coil Domain

**DOI:** 10.3390/biom8040162

**Published:** 2018-12-04

**Authors:** Andrea M. Clark, Komala Ponniah, Meghan S. Warden, Emily M. Raitt, Andrea C. Yawn, Steven M. Pascal

**Affiliations:** Department of Chemistry and Biochemistry, Old Dominion University, Norfolk, VA 23529, USA; akorell@odu.edu (A.M.C.); kponniah@odu.edu (K.P.); mwarden@odu.edu (M.S.W.); erait001@odu.edu (E.M.R.); ayawn@odu.edu (A.C.Y.)

**Keywords:** prostate apoptosis response-4 (Par-4), intrinsically disordered protein (IDP), cancer, circular dichroism, coiled coil (CC), leucine zipper (LZ), apoptosis

## Abstract

Prostate apoptosis response-4 (Par-4) is a 38 kDa largely intrinsically disordered tumor suppressor protein that functions in cancer cell apoptosis. Par-4 down-regulation is often observed in cancer while up-regulation is characteristic of neurodegenerative conditions such as Alzheimer’s disease. Cleavage of Par-4 by caspase-3 activates tumor suppression via formation of an approximately 25 kDa fragment (cl-Par-4) that enters the nucleus and inhibits Bcl-2 and NF-ƙB, which function in pro-survival pathways. Here, we have investigated the structure of cl-Par-4 using biophysical techniques including circular dichroism (CD) spectroscopy, dynamic light scattering (DLS), and intrinsic tyrosine fluorescence. The results demonstrate pH-dependent folding of cl-Par-4, with high disorder and aggregation at neutral pH, but a largely folded, non-aggregated conformation at acidic pH.

## 1. Introduction

Par-4 is a pro-apoptotic tumor suppressor protein that was first identified in studies of prostate cancer [1,2,3]. In normal mammalian tissue, Par-4 is ubiquitously expressed, localized in the cytoplasm of healthy cells, and spontaneously secreted [1,2,4,5]. Par-4 down-regulation has been implicated in a variety of cancers including prostate, renal, breast, endometrial, leukemia, and neuroblastoma [6,7,8,9]. Furthermore, low Par-4 levels correlate to metastasis and an increased chance of cancer recurrence [8,10]. The *Par-4/PAWR* gene is found on the unstable chromosome 12q21.2, which is often mutated or deleted in cancers such as gastric and pancreatic [11].

The most well characterized mode of cancer cell apoptosis induced by extracellular Par-4 is mediated via interaction of Par-4 with the cell surface GRP78 protein [4]. This interaction initiates the apoptotic Fas/FasL-FADD caspase-8 pro-death pathway [4,12,13]. Healthy cells have low cell surface levels of GRP78, providing resistance to extracellular Par-4-induced apoptosis [4,14]. Par-4 can also induce cancer cell apoptosis intracellularly via nuclear translocation of a C-terminal fragment, as discussed further below [10,15,16].

Par-4 contains two nuclear localization sequences (NLS1 and NLS2), a VASA domain (a sequence similar to that found in the VASA protein), a selective for apoptosis induction in cancer cells (SAC) domain, a coiled coil (CC) domain with a leucine zipper (LZ), and a nuclear export sequence (NES) [2,5,17]. The SAC domain is the minimum fragment necessary to induce apoptosis [17,18,19]. The C-terminal CC plays a role in homo- and hetero-dimerization and regulation of Par-4 function [2,18,20,21,22,23].

The Par-4 CC contains approximately 78 amino acids, with the C-terminal half of the CC displaying a marked heptad repeat characteristic of a LZ [5]. The isolated C-terminal LZ can interconvert between a partially ordered monomer (POM) and a coiled coil dimer (CCD) [21,22]. The LZ associates tighter under acidic conditions, forming a more stable dimer [24,25]. Dimer formation in the entire CC domain was confirmed by x-ray crystallography [23]. However, little is known about the structure of the remainder of the protein except that most of full-length Par-4, outside of the CC, appears to be disordered in vitro [21,22]. Intrinsically disordered proteins (IDPs) lack a stable structure and many disease-associated proteins can be classified as IDPs [26,27,28,29].

**Figure 1:**
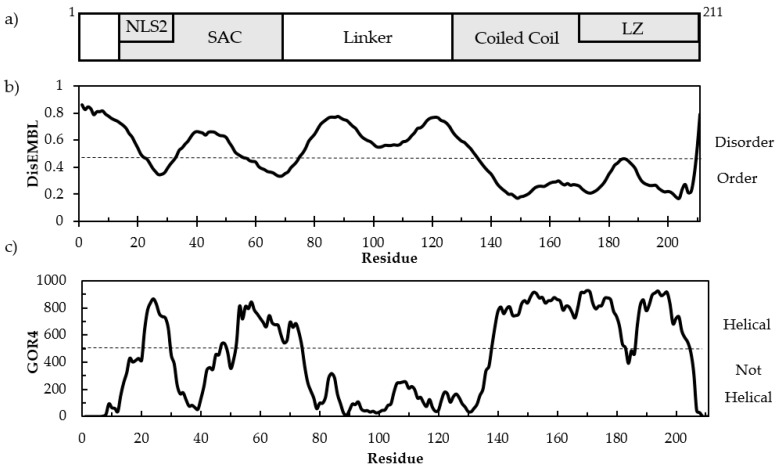
(**a**) Block diagram of caspase-3-cleaved Par-4 (cl-Par-4) domain structure. (**b**) DisEMBL disorder prediction. (**c**) GOR4 (Garnier-Osguthorpe-Robson) alpha helix prediction.

Caspase-3-mediated cleavage of Par-4 at Asp-131 generates two fragments: a 15 kDa amino-terminal fragment (“PAF” Par-4 Amino terminal Fragment) and a 25 kDa C-terminal fragment, cleaved Par-4 (cl-Par-4) [30,31]. The PAF contains the NLS1 and VASA segment and remains in the cytoplasm [17,32,33,34,35]. cl-Par-4 retains the SAC domain (residues 16–71) that includes NLS2, the CC with LZ (133–211), and a linker domain (72–132) connecting the SAC and CC domains, where numbering is in the context of the fragment (Figure 1a) [17,30]. cl-Par-4 translocates to the nucleus via NLS2 [5], where it inhibits pro-survival pathways that mediate cancer cell survival including NF-ƙB and Bcl-2 [13,16,17,30,34,36]. cl-Par-4 is an attractive therapeutic target because of its potential to specifically target cancer cells.

Here, we have studied the conformation of cl-Par-4 in vitro. Results show that at neutral pH, cl-Par-4 forms partially folded, soluble aggregates with a high degree of conformational flexibility. In contrast, at acidic pH, cl-Par-4 adopts a predominantly folded, stable, largely alpha helical conformation. While the CC has been previously structurally characterized, this is the first evidence of structure outside of the CC domain in Par-4. These observations have implications for understanding cl-Par-4 structure and function in relation to cellular localization.

## 2. Materials and Methods

### 2.1. Expression and Purification of cl-Par-4

The cl-Par-4 construct (residues 132–340 of human Par-4) was prepared by PCR amplification with the following primers: 5’-GACCCATGGGTGTTCCGGAGAAGGGCAAAAGC-3’ (forward) and 5’-CAGAAGCTTTTAGCGGGTCAGTTGGCCCACCAC-3’ (reverse) using full length Par-4 as a template. PCR primers were purchased from Eurofins Genomics (Louisville, KY, USA). The PCR product was cleaved with NcoI/HindIII and ligated into a modified version of the expression vector H-MBP-3C [37]. DNA sequence of the construct was verified through sequencing and protein expression was carried out in BL21 (DE3) CodonPlus cells. The cells were grown in Luria-Burtani (LB) media with 100 μg/mL ampicillin at 37 °C until an OD_600_ of 0.8–0.9 was reached. Protein expression was induced with the addition of 0.5 mM isopropyl thio-β-D-galactoside (IPTG) and then grown for an additional 18 h at 15 °C.

The cells were harvested by centrifugation and the resulting cell pellet was resuspended in lysis buffer (10 mM Tris, 300 mM NaCl, 20 mM imidazole, 1 mM TCEP, pH 7.4) containing 1 mg/mL lysozyme. Cells in lysis buffer were sonicated with a 10 s pulse/59 s rest at 40% amp for 30 repetitions, followed by centrifugation at 16,000 rpm. The lysate was filtered through a 0.8 μm syringe filter, followed by filtration with a 0.45 μm filter. The clarified cell lysate was then purified with IMAC using a His-Trap HP column (GE Healthcare, Uppsala, Sweden) and eluted with buffer containing 300 mM imidazole. Fractions containing the protein of interest were pooled and dialyzed in 3C protease cleavage buffer (10 mM Tris, 1 M NaCl, 1 mM TCEP, pH 7.5). The His-MBP tag was removed by cleavage with 3C protease at 4 °C in 3C protease cleavage buffer. Cleavage left a two-residue remnant (Gly-Pro) at the N-terminus of cl-Par-4. As a final purification step the cleaved product was dialyzed against 10 mM Tris, 1 M NaCl, 20 mM imidazole and 1 mM TCEP, pH 8.0 and loaded onto a His-Trap HP column to remove the tag.

Purified cl-Par-4 was dialyzed against 10 mM Tris, 1 M NaCl, 1 mM TCEP, pH 7.0, and then concentrated by centrifugation at 3500 rpm using a Vivaspin Turbo 15 (Sartorius, Epsom, UK). Absorbance measurements at 280 nm were taken to determine protein concentration using the extinction coefficient 6400 M^−1^cm^−1^. Purified cl-Par-4 was lyophilized in 10 mM Tris, 1 M NaCl, 1 mM TCEP, pH 7.0, and re-solubilized in sterile distilled H_2_O. TCEP was used in the purification steps to prevent aggregation via disulfide bond formation.

The theoretical molecular weight of the bacterially expressed cl-Par-4 used for this study was 24 kDa, but has been approximated as 25 kDa throughout this manuscript for consistency with prior literature, where the molecular weight was determined via Western blots performed with mammalian extracts [30].

### 2.2. Circular Dichroism Measurements

Circular dichroism (CD) spectra were recorded on a J-815 CD spectrometer (Jasco, Easton, MD, USA). Samples were at a concentration of 0.2 mg/mL in native buffer (20 mM NaCl, 10 mM Tris, 1 mM TCEP) and native buffer with 0.1% SDS, ranging from pH 4–10. Far UV-CD spectra were recorded from 260–190 nm at a scan speed of 20 nm/min with a bandwidth of 1 nm. Samples were recorded at 25 °C unless otherwise noted. Thermal stability was probed by recording CD spectra at 5, 25, 45, 65, and 85 °C. Three scans were recorded for each sample and then averaged after baseline subtraction. The scans were smoothed using a means-movement function of 25. CD spectra were deconvoluted using the Selcon3 algorithm through the DichroWeb server [38].

### 2.3. Dynamic Light Scattering and Zeta Potential Measurements

DLS measurements were recorded using a NanoBrook Omni particle sizer and zeta potential analyzer (Brookhaven Instruments Corporation, Holtsville, NY, USA). Samples were at a concentration of 0.2 mg/mL in native buffer over a pH range from 4–10. DLS data was obtained at 25 °C using 1 cm path length plastic cuvettes. Five scans were recorded and averaged for each sample. The highest peak of the histogram was recorded as the mean diameter for that sample and the hydrodynamic radius was calculated using the Stoke-Einstein equation. The cl-Par-4 zeta potential measurements were also recorded at a concentration of 0.2 mg/mL in native buffer over pH 4–10. Five measurements in mV were recorded and averaged for each pH, and the experimental pI was determined.

### 2.4. Fluorescence Spectroscopy

Fluorescence measurements were recorded using a Varian Cary Eclipse Fluorescence Spectrophotometer (Varian Inc., Santa Clara, CA, USA). Samples were at a concentration of 0.2 mg/mL in native buffer at pH 4 and 7 in 1 cm path length, 400 μL fluorescence cuvettes. Tyrosine was selectively excited at 220 nm (determined as the maximum excitation wavelength) and the emission spectrum was recorded over 250–400 nm. An excitation slit width of 10 nm and an emission slit width of 20 nm was used. Three scans were recorded and averaged after baseline subtraction for each sample at 25 °C. For thermal denaturation studies, the fluorescence intensity at 310 nm was recorded from 20–95 °C with a temperature rate increase of 1 °C/min using the same excitation and slit parameters as above.

## 3. Results

### 3.1. Sequence Analysis and Structure Predictions

Disorder prediction in cl-Par-4 was performed using DisEMBL analysis (Figure 1b). Disorder probability greater than 0.43 (dashed line) approximately separates order and disorder [39]. The data show high disorder probability in the linker domain with mixed order and disorder in the SAC domain. The region with the most order aligns with the CC and LZ.

Secondary structure prediction was performed using GOR4 analysis, which predicted 48.8% helical content, 41.7% random coil, and 9.5% extended strand in cl-Par-4 [40]. Figure 1c shows alpha helix prediction using GOR4 (Garnier-Osguthorpe-Robson) analysis. Some helical propensity occurred in the SAC domain, while very low helicity was predicted in the linker domain. The highest helical propensity occurs in the CC and LZ. However, between residues 180–190, a decrease in helical propensity could be seen. This decrease corresponds to the position of a charge–charge repulsion across the CC dimer interface [24,25], as will be discussed further below. The regions with high disorder probability by DisEMBL aligns with regions of low helical propensity by GOR4.

At neutral pH, cl-Par-4 is comprised of 19.9% negatively charged amino acids and 18.5% positively charged residues. This is significantly higher than the observed averages for vertebrate proteins: 11.7% and 11.4% for negatively and positively charged residues, respectively [41,42]. In general, high charge content in IDPs can contribute to the formation of extended conformations that minimize electrostatic repulsion between like charges [28]. Sodium dodecyl sulfate polyacrylamide gel electrophoresis (SDS-PAGE) of cl-Par-4 displays an apparent molecular weight of 31 kDa, approximately 30% higher than the expected molecular weight of 25 kDa (Figure S1). Differential SDS-binding coupled with the fully extended conformation of IDPs can cause slower electrophoretic mobility [26,43,44].

### 3.2. Secondary Structure Characterization by CD Spectroscopy

**Figure 2:**
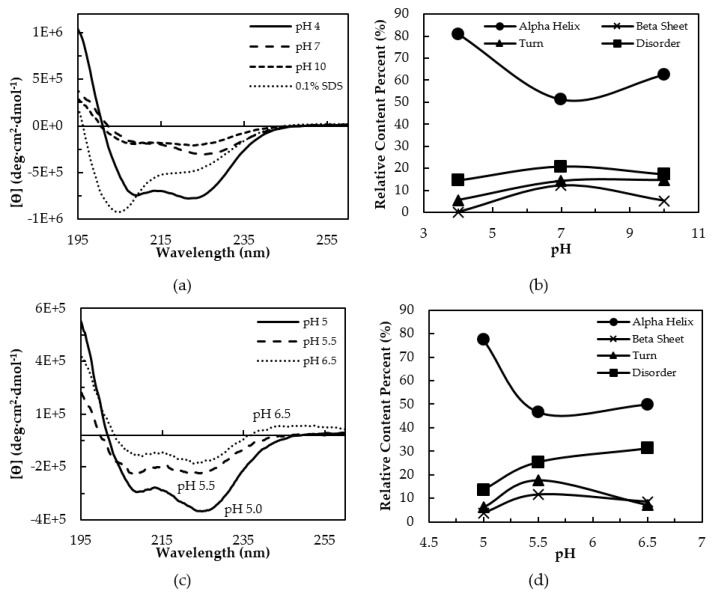
(**a**) Circular dichroism (CD) analysis of cl-Par-4 at pH 4, 7, 10, and in SDS (sodium dodecyl sulfate). (**b**) Secondary structure content at pH 4, 7 and 10. (**c**) CD analysis at pH 5, 5.5, and 6.5, near the pI. (**d**) Secondary structure content near the pI.

The secondary structure of cl-Par-4 was investigated using CD spectroscopy under varying pH conditions (Figure 2a). A pair of minima at 222 nm and 208 nm are characteristic of alpha helical secondary structure. These minima were observed both under neutral conditions (pH 7) and more extreme conditions (pH 4 and pH 10). However, the CD spectrum at pH 4 showed significantly more alpha helical character with intense negative transitions at these wavelengths. Helical content was lower at pH 7 and 10, as indicated by less intense dichroism at both 222 nm and 208 nm. The CD spectra of cl-Par-4 was also examined under denaturing conditions of 0.1% SDS to assess the mostly unfolded state for comparison. With 0.1% SDS, an intense minimum at 205 nm dominated the spectrum, though a second feature appeared near 222 nm. This spectrum is characteristic of a mostly disordered conformation with a degree of residual secondary structure.

Consistent with the two intense minima at pH 4, spectral deconvolution indicated an alpha helical content of 81%, with a 14% disorder (Figure 2b). Beta sheet or turn content was minimal. At neutral pH, calculations showed cl-Par-4 to be only about ½ alpha helical, with 30% beta content (sheet + turn) and 22% disorder. At pH 10, an intermediate level of alpha helix (62%) with 15% turn and 17% disorder was calculated. The unfolded state in SDS was calculated to possess three approximately equal parts of 1/3 alpha helical character, 1/3 beta character, and 1/3 disorder.

A ratio of ϴ_222_/ϴ_208_ greater than 1 is characteristic of CC formation [45]. Ratios were 1.2, 1.6, and 1.1 at pH 4, 7, and 10, respectively (Table S1), and each suggest some level of dimeric association of helices. The high ratio at pH 7 could be influenced by the increased level of beta secondary structure, which can augment the dichroism at 222 nm. Under SDS denaturing conditions, the ϴ_222_/ϴ_208_ ratio was 0.60, which was not suggestive of CC formation, and disorder was increased. Together, the data suggest that low pH induces a well-folded CC and possibly additional non-coiled helices, while pH 7 and 10 also induce some CC formation, but with additional beta-like and disordered content.

The CD spectra in Figure 2a show a marked difference in secondary structure above vs. below the cl-Par-4 theoretical isoelectric point (pI) of 5.39. To further investigate the conformation near the pI, additional CD spectra were recorded at pH 5.0, 5.5, and 6.5 (Figure 2c), with secondary structure deconvolution charted in Figure 2d. Data were not acquired at pH 6.0 due to sample aggregation. Below the pI at pH 5.0, a characteristic alpha helical spectrum indicated an approximately 80% helical content, with a ϴ_222_/ϴ_208_ ratio of 1.3, consistent with the formation of CC (Table S2). In contrast, the spectra recorded above the pI both indicated approximately 50% helix. A lower ϴ_222_/ϴ_208_ ratio of 1.0 was observed at pH 5.5, which was close to the pI. The ratio was 1.3 at pH 6.5; however, as discussed above, increased levels of beta secondary structure could affect this ratio. These results confirm additional helicity induced by pH below the pI value.

**Figure 3:**
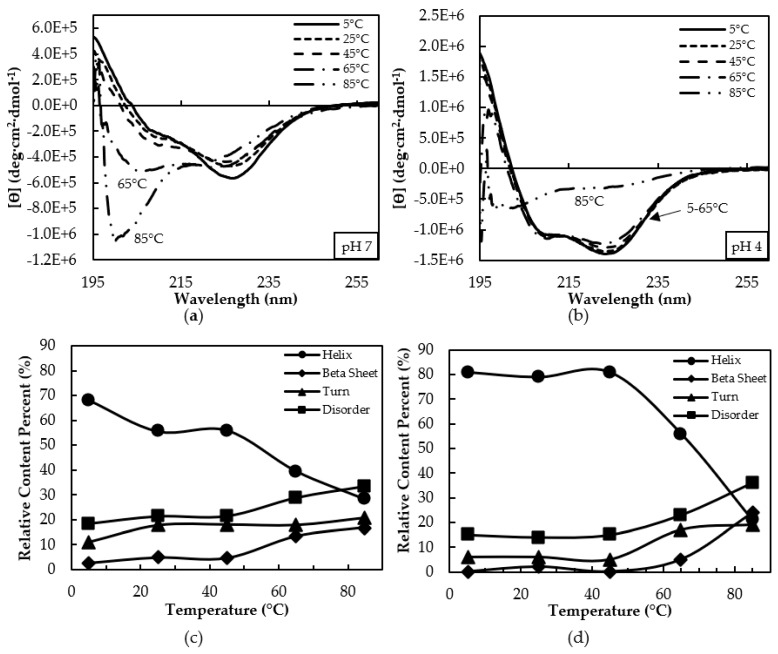
(**a**) Circular dichroism (CD) versus temperature at pH 7. (**b**) CD versus temperature at pH 4.(**c**) Secondary structure versus temperature at pH 7. (**d**) Secondary structure versus temperature at pH 4.

Next, the thermal dependence of the Far UV-CD spectrum at pH 7 and pH 4 were compared (Figure 3). At 5 °C, the CD spectra at pH 7 showed an intense minimum near 225 nm, but less intensity near 208 nm (Figure 3a). The spectral changes were modest through to 45 °C, then a major change occurred at 65 °C and 85 °C, with an intense band arising near 200 nm, characteristic of disorder. In contrast, spectra at pH 4 showed more alpha helical content, with intense minima at both 208 and 222 nm (Figure 3b). The spectra at pH 4 was consistent with a predominantly folded, alpha helical conformation even up to 65 °C, with significant disorder only arising at 85 °C.

At neutral pH and 5 °C, there was approximately 2/3 calculated helical content with 20% disorder (Figure 3c). Between 25–45 ºC, helical content decreased to approximately 1/2 with a corresponding increase in beta content (sheet + turn) to approximately 20%. At 65 °C, helicity decreased to 40% with increased beta content to 31% and disorder to almost 30%. At 85 °C, helicity decreased to approximately 1/4 with an increase in beta content to 40%, and 34% disorder. The ϴ_222_/ϴ_208_ ratios were greater than 1 at temperatures up to 45 °C (Table S3), but these values may have been influenced by the presence of the beta secondary structure. Ratios were less than 1 at temperatures above 65 °C.

At acidic pH, approximately 80% helical content with marginal disorder was observed from 5 to 45 °C, with decreased helical content and concomitant increase in beta and disorder content seen at 65 °C, although the change in spectral appearance seems relatively minor (Figure 3d). The major spectral change at 85 °C corresponds to a helicity reduction to approximately 20% with increased beta and disorder contents to approximately 40% each. The ϴ_222_/ϴ_208_ ratios suggest CC formation at least up to 65 °C, which was lost by 85 °C (Table S4).

### 3.3. Dynamic Light Scattering and Zeta Potential

**Figure 4:**
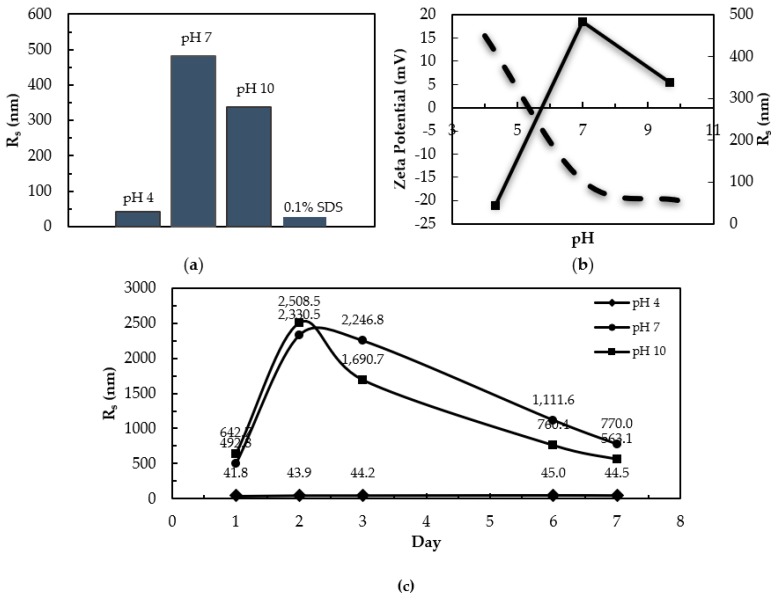
(**a**) Measured R_s_ of cl-Par-4 under native and denaturing conditions by Dynamic Light Scattering (DLS). (**b**) Relationship of zeta potential (dashed) to pH and R_s_ (solid line). (**c**) Measured R_s_ over seven days at pH 4, 7, and 10.

Dynamic light scattering (DLS) was used to assess the size and aggregation state of cl-Par-4 under varying conditions. The measured stokes radius (R_s_) values were 483 nm at pH 7 and 339 nm at pH 10, while measurements at pH 4 gave an R_s_ of 43 nm (Figure 4a). DLS measurements were also recorded under denaturing conditions of 0.1% SDS at pH 7, producing an R_s_ of 28.3 nm. The experimental pI was determined by zeta potential measurements (Figure 4b). Zeta potential decreased from +15.4 to −20.01 mV when pH was increased from 4–10. The experimental pI was determined to be pH 5.35, consistent with the theoretical pI of 5.39. Figure 4c shows the R_s_ measurements over the course of seven days at pH 4, 7, and 10. At pH 7 and 10, the R_s_ changed substantially over seven days with significant variation in measured R_s_. There was little variation in acidic conditions, with R_s_ ranging from 41.8–45.0 nm over time.

The R_s_ values at pH 7 and 10 indicate aggregation above the pI of 5.35. A large R_s_ can also be indicative of a non-globular or rod-shaped conformation. However, the values here were much larger than expected for a monomeric or dimeric rod. In contrast, the R_s_ value at pH 4 did not indicate the presence of large aggregates, and data at acidic pH were more monodisperse and time-stable than at neutral pH, although the R_s_ was still larger than for the sample in SDS. In fact, the Stokes radii of all non-denatured samples, from pH 4–10, were larger than the partially unfolded SDS form, suggesting a polymeric state for cl-Par-4 under each of these conditions. At least some of this self-association is mediated by the C-terminal CC dimerization motif.

### 3.4. Tertiary Structure by Fluorescence

**Figure 5:**
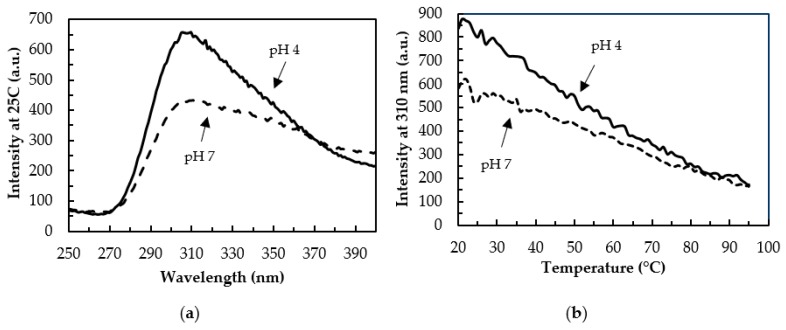
(**a**) Intrinsic tyrosine fluorescence at pH 4 (solid) and 7 (dashed) over 250–400 nm at 25 °C (**b**) Thermal denaturation at pH 4 (solid) and 7 (dashed) monitored by fluorescence emission at 310 nm.

Intrinsic fluorescence can be used to monitor the solvent exposure of aromatic amino acids and so provide information regarding tertiary structure [46]. cl-Par-4 has one tyrosine in the SAC domain and four tyrosines in the linker domain. Interestingly, these regions are predicted to be disordered based on DisEMBL and GOR4 analysis [39,40]. Tyrosine was selectively excited at 220 nm. Figure 5a shows the resulting tyrosine emission spectrum from 250–400 nm at 25 °C for samples at pH 7 and pH 4. The emission maximum near 310 nm was considerably more intense at pH 4 than at pH 7.

Thermal denaturation at pH 7 and pH 4 was also investigated (Figure 5b) by monitoring the tyrosine emission at 310 nm as the temperature was increased from 20–95 °C. Both samples showed a marked decrease in fluorescence emission with rising temperature. Together, these spectra indicate that at acidic pH, cl-Par-4 adopts a fold that protects the tyrosines from solvent exposure, more so than the conformation at neutral pH. However, under both conditions, substantial solvent protection of tyrosine residues exists at low temperatures.

## 4. Discussion

### 4.1. Intrinsic Disorder in cl-Par-4

Disorder-to-order transition plays a major role in IDP functions, such as the regulation of cell signaling and ligand binding [27,47]. Common features of IDPs include reduced sequence complexity, high net charge, and often increased stability at extreme temperature and pH [26,47,48]. This last feature is clearly related to the high content of charged residues, since extreme pH will change the charge distribution. In most cases, this will reduce the number of charged side chains, thus reducing charge–charge repulsions that inhibit folding of the protein.

Full-length Par-4 has been classified as mostly intrinsically disordered, although it contains a helical CC at its C-terminus [21,22,23]. However, it has been established that apoptosis is directly triggered by the 25 kDa cl-Par-4 fragment, which is capable of entering the nucleus [30]. This “activated” fragment of Par-4 was studied here, bearing in mind that it derives from the largely disordered full-length protein. DisEMBL disorder prediction showed a high disorder probability in the linker domain and some disorder in the SAC domain, which coincided with regions of low helix propensity by GOR4 analysis (Figure 1b,c) [39,40]. Additionally, GOR4 analysis predicted approximately 42% disorder under physiological conditions (Figure 1c) [40].

Consistent with this prediction, we found evidence for disorder in cl-Par-4. First, cl-Par-4 displayed an apparent molecular weight of 31 kDa via SDS-PAGE analysis, which was approximately 30% higher than expected (Figure S1). This was consistent with previous studies on the racine Par-4 full length, SAC, and deleted LZ constructs, which each showed apparent molecular weights based on SDS-PAGE, which were at least 30% higher than the predictions based on the primary structure and experimentally determined via mass spectroscopy [21]. This behavior is due to the unique negatively-charged amino acid composition typical of IDPs, which reduces the affinity of SDS binding, preventing full denaturation and decreasing electrophoretic mobility [26,43,44]. CD (Figure 2a, Table S1) and DLS (Figure 4a) analysis were also consistent with only partial disorder in the presence of SDS. Thermal denaturation (Figure 3, Tables S2 and S3) was better able to unfold cl-Par-4, confirming that a significant degree of structure remains in the presence of the SDS denaturant.

### 4.2. Instability at Neutral pH

At neutral pH, cl-Par-4 shows a mix of order and disorder. CD spectra at pH 7 shows cl-Par-4 to be approximately 50% helical, 30% beta, and 20% disordered (Figure 2) with significant temperature sensitivity (Figure 3). DLS experiments showed large measured R_s_, which varied with time. IDPs have a larger R_s_ than a globular protein of the same molar mass. However, the R_s_ value here was far larger than expected for a disordered monomer, and instead indicates aggregation [49,50]. Fluorescence emission spectra indicated the partial protection of tyrosine residues in the SAC and linker domains.

A high ϴ_222_/ϴ_208_ ratio indicates CC formation, and hence self-association, although beta content could inflate this ratio. Conformational instability and increased size occurred at all pH values tested above the experimentally determined pI of 5.35 (Figure 4b). CD spectra showed significantly more helical content at pH 5 than at pH 5.5 or pH 6.5 (Figure 2c). Taken together, the data demonstrated that at pH above the pI, aggregation, conformational instability, and partial disorder resulted.

**Figure 6:**
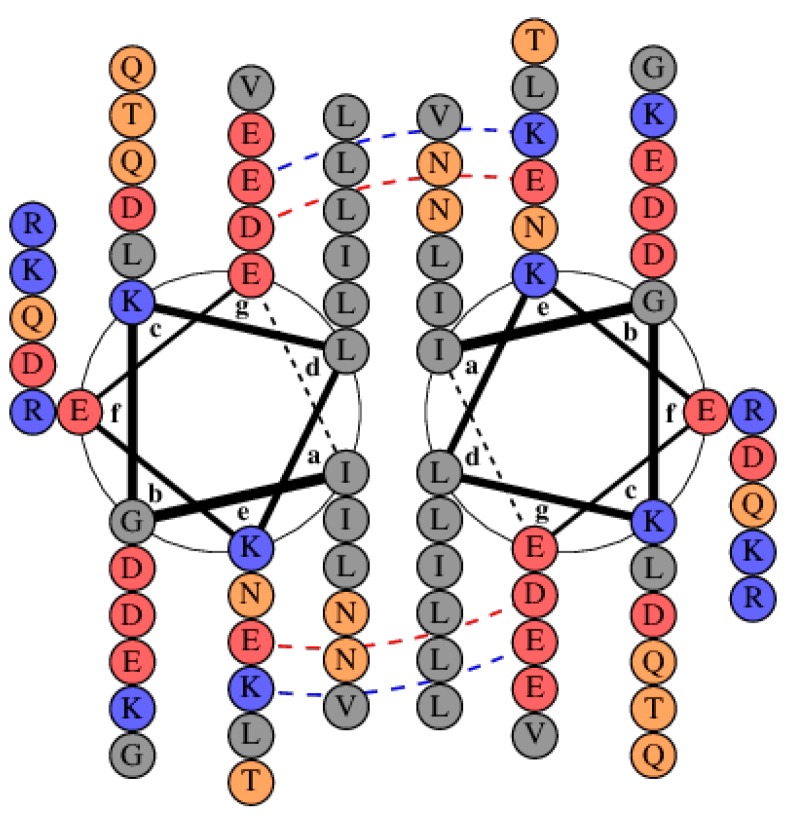
Helical wheel representation of Par-4 leucine zipper parallel dimer (DrawCoil 1.0, Dartmouth College, Hanover, NH, USA). Basic residues are blue and acidic residues are red. The red dashed line represents inter-helical charge repulsion and the blue dashed line represents an inter-helical salt bridge.

Figure 6 shows a helical wheel diagram of the LZ region of Par-4. LZs are a special type of coiled-coil oligomerization motif with leucines in the *d* position [51,52,53]. The dashed lines in Figure 6 represent inter-helical interaction between charged residues at the *e* and *g* positions. The D-E charge repulsion contributes to the conformational instability observed above the pI, as previously determined for the LZ and CC constructs [22,24,25]. In summary, the disorder and aggregation at pH above the pI can be explained via electrostatic repulsion across the LZ dimer interface.

### 4.3. Acidic pH Induces folding

Negative–negative electrostatic repulsion within the cl-Par-4 CC domain is abrogated at low pH due to partial titration of the acidic side chains involved [24,25]. Due to the associating negative charges, the pKas of the repelling D-E acidic side chains (Figure 6) in the CC domain are expected to be higher than normal and should be somewhere in the range of 5–6. This was consistent with the increased stability seen at pH 5 vs. pH 5.5 and pH 6.0.

It is for this reason that, at pH below the pI, the cl-Par-4 helical content was high (approximately 80%), and CC formation was evident from ϴ_222_/ϴ_208_ ratios, particularly at pH 5 (Figure 2c). DLS showed a monodisperse conformation with an R_s_ value intermediate between the largely disordered monomer in SDS and the half-disordered aggregate at neutral pH (Figure 4). Fluorescence showed significant protection of the tyrosine residues in the SAC and linker domains (Figure 5). Together, these data suggest that at acidic pH, cl-Par-4 forms a single, stable conformation of fixed self-association state, with substantial CC and perhaps additional non-coiled helical regions. The amount of helix present requires that at least part of the SAC and linker domains have a helical character.

While CD spectroscopy indicates thermal stability up to at least 65 °C, the 222 nm band became systematically less intense with increasing temperature (Figure 3b). This suggests a reduction in CC content at higher temperatures (ϴ_222_/ϴ_208_ ratios in Table S4). Some IDPs such as nerve growth factor and αs-casein gain structure upon increased temperature [54,55]. Our results showed the opposite trend: increased temperature resulted in the partial loss of secondary structure in cl-Par-4, providing further evidence of an ordered structure.

**Figure 7:**
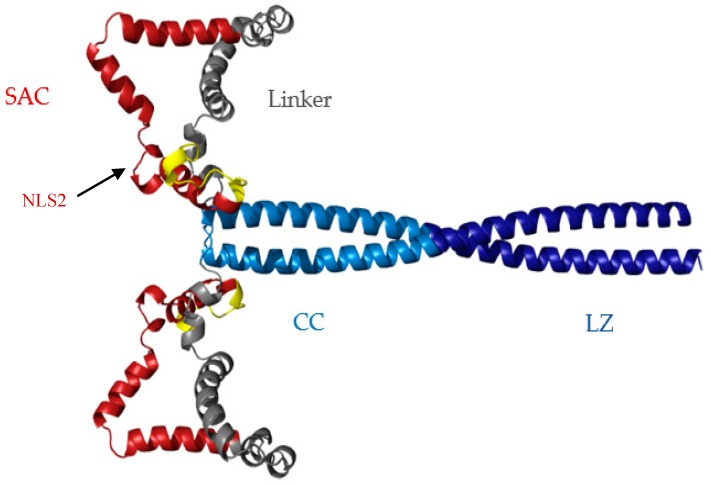
GalaxyWEB model of cl-Par-4 at acidic pH. The N-terminal residues are yellow, the selective for apoptosis induction in cancer cells (SAC) domain with nuclear localization signal 2 (NLS2) is red, the linker region is gray, and coiled coil (CC) is blue. Darker blue is the leucine zipper (LZ) region of the CC. The position of NLS2 is also indicated.

To further assess the conformation at acidic pH, template-based models were generated via GalaxyTBM on the GalaxyWEB server [56]. The racine Par-4 CC crystal structure (pdb 5fiy_A) was used as a template and the remainder of the protein was allowed to fold computationally [23]. An ensemble of five structures was generated and visually inspected for features consistent with the above biophysical results for cl-Par-4 at acidic pH. At least part of the SAC and linker domains must fold under acidic conditions since the CC only comprised approximately 37% of the protein fragment, while CD analysis indicated approximately 80% helical content. The structure shown in Figure 7 is representative of the type of conformation that may occur at acidic pH: a relatively compact conformation with partially helical SAC and linker domains attached to the C-terminal CC.

Interestingly, increased caspase-3 activation is known to occur during apoptosis due to the release of cysteine proteases from the lysosome, during cytosolic acidification [57,58,59,60,61]. This suggests that cl-Par-4 may be formed under acidic conditions. For subsequent nuclear entry, another protein such as a nuclear import receptor must bind the NLS of cl-Par-4 [5,62,63]. The NLS would likely be accessible in a folded non-aggregated conformation such as that represented in Figure 7, but may not be accessible in the largely disordered aggregate present at neutral pH.

Consistent with this possible mechanism, the Ras association domain family member 2 (RASSF2) tumor suppressor has been shown to bind a cl-Par-4-sized fragment of Par-4, via the NLS [64]. This binding interaction enhances the nuclear localization of the Par-4 fragment, leading to cancer cell apoptosis [64]. In contrast, the NES is most likely masked by homo-dimerization mediated by the CC, preventing the nuclear exit of cl-Par-4 [12]. Taken together, these results indicate that caspase-induced cleavage of Par-4, creating cl-Par-4, simultaneously exposes the cl-Par-4 NLS and sequesters the nuclear export sequence (NES), obligating nuclear localization. The folded conformation shown here is consistent with exposure of the NLS at its N-terminus, and sequestration of the NES within the CC dimerization domain. Furthermore, we have shown that at acidic pH, aggregation of cl-Par-4 is inhibited, producing a molecular size more consistent with the ability to traverse nuclear pores.

### 4.4. Acidic Environments

Though acidic pH can induce cl-Par-4 folding in vitro, the physiological relevance of the acid-induced structure of cl-Par-4 remains to be determined. Some clues to the importance of folding at acidic pH can be obtained through discussion of other proteins with similar characteristics.

First, many in vitro studies have shown IDPs preferentially folding at acidic pH [65,66]. Examples include α-synuclein, prothymosin α, and the cytoplasmic domain of BAP29 [65,67,68,69]. The general principle is that acidic pH can alleviate charge–charge repulsion in IDPs, allowing for stable folding [26,28,47]. Other proteins are known to both have a stable structure and to function in an acidic environment. For instance, acid endonucleases function in cell death and have optimal activity as low as pH 4.9 [70,71]. As a second example, dimer formation of the apoptosis-regulating Bcl-2 family proteins is stabilized at pH 4 [72].

Many other proteins, including tumor suppressors, have been detected in lysosomes, endosomes or exosomes, which can be highly acidic. Perhaps the best known example is the p53 tumor suppressor, which can be found in highly acidic lysosomes in human breast cancer cells [66,73]. It has been suggested that the p53 conformation at acidic pH may function in lysosomal membrane permeabilization, which often occurs in early apoptosis [66,74]. The PTEN protein is an example of a tumor suppressor that is transported via exosomes [75]. Exosomes can be used for the intercellular transfer of tumor suppressors, which helps to prevent tumor proliferation [76,77]. Exosome biogenesis occurs through the lysosome–endosome pathway (pH 4–6 range), a pH range that is consistent with the acidic pH in our study [78,79,80,81,82,83,84].

Therefore, it is interesting to note that while most studies of Par-4 have focused on intracellular pathways, full length Par-4, along with Par-4 fragments of 33 and 14 kDa (based on SDS-PAGE) have also been found in secreted exosomes (termed apoxosomes) [85]. These fragments have not yet been positively identified, however, the SDS-PAGE-based sizes are consistent with those created by caspase-induced cleavage, including the cl-Par-4 fragment. Additionally, p62 forms a ternary complex with PKCζ (protein kinase C) and Par-4 (through the Par-4 C-terminus), helping to regulate the NF-κB pathway [86]. Interestingly, atypical isoforms of PKC, such as PKCζ, co-localize with p62 to late endosomal compartments [86,87,88,89], suggesting that Par-4 may also localize to late endosomes. Therefore, it is quite plausible that future studies will positively identify cl-Par-4 in these acidic organelles. Additionally, the fact that the typical exosome size is less than 100 nm [90] suggests that cl-Par-4 under neutral conditions (R_s_ > 400 nm) may be too large for exosomal transport. However, the folded conformation at acidic pH would be of a more suitable size. It has also been well documented that the cytosol of cancer cells can become acidic, particularly during apoptotic processes [60,61,70,91,92,93,94,95]. For instance, one study showed apoptotic human histiocytic lymphoma cells with a cytosolic pH of 5.7 [73,81]. While the degree of acidification or alkalination may vary in different tumors, cancer-related acidification of the cytosol, when it does occur, could potentially help to promote IDP folding.

Finally, it should be noted that in vivo folding of cl-Par-4 may be influenced by factors other than pH, including post-translational modification or interactions with other proteins or ions. This could help to reduce the conformational instability observed at neutral pH. However, these factors are not required for the stable folding of cl-Par-4 at acidic pH.

## 5. Conclusions

In summary, the data in the present study demonstrate that folding of the cl-Par-4 tumor suppressor is markedly pH-dependent: cl-Par-4 is partially folded and aggregated at neutral pH, but folds into a non-aggregated, mostly helical conformation at acidic pH. This is the first evidence of structure outside of the CC domain in Par-4, indicating that cl-Par-4, under acidic conditions, should be classified as an ordered, folded protein.

## Supplementary Materials

The following are available online at http://www.mdpi.com/2218-273X/8/4/162/s1. Figure S1: SDS-PAGE of cl-Par-4 (25kDa); Table S1: Secondary structure percentages of cl-Par-4 under at pH 4, 7, 10, and SDS denaturing conditions by deconvolution of the CD spectra; Table S2: Secondary structure percentages of cl-Par-4 near the pI at pH 5, 5.5, and 6.5 by deconvolution of the CD spectra; Table S3: Thermal stability of cl-Par-4 at neutral pH by deconvolution of the CD spectra; Table S4: Thermal stability of cl-Par-4 at acidic pH by deconvolution of the CD spectra.
