# Supplementary material for: pH-Induced Folding of the Caspase-Cleaved Par-4 Tumor Suppressor: Evidence of Structure Outside of the Coiled Coil Domain
Source: Biomolecules. 2018 Dec 4;8(4):162. doi: 10.3390/biom8040162 (PMC6316887; doi:10.3390/biom8040162)
Supplement: Supplementary file 1 [file biomolecules-08-00162-s001.docx]

**Supplementary Materials:**


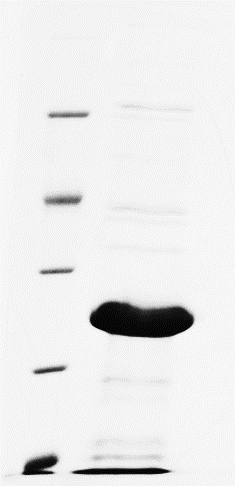


116.0

66.2

45.0

35.0

25.0

18.4

**Figure S1.** SDS-PAGE gel electrophoresis of cl-Par-4 (25kDa).

**Table S1.** Secondary structure percentages of cl-Par-4 under at pH 4, 7, 10, and SDS denaturing conditions by deconvolution of the CD spectra.

| **pH** | **Helix (%)** | **β-Sheet (%)** | **Turn (%)** | **Disorder (%)** | **ϴ_222_/ϴ_208_** |
| --- | --- | --- | --- | --- | --- |
| 4 | 81 | 0 | 5 | 14 | 1.2 |
| 7 | 47 | 15 | 14 | 22 | 1.6 |
| 10 | 62 | 5 | 14 | 17 | 1.1 |
| 7, 0.1% SDS | 36 | 11 | 24 | 32 | 0.6 |

**Table S2.** Secondary structure percentages of cl-Par-4 near the pI at pH 5, 5.5, and 6.5 by deconvolution of the CD spectra.

| **pH** | **Helix (%)** | **β-Sheet (%)** | **Turn (%)** | **Disorder (%)** | **ϴ_222_/ϴ_208_** |
| --- | --- | --- | --- | --- | --- |
| 5 | 78 | 4 | 6 | 14 | 1.3 |
| 5.5 | 47 | 12 | 18 | 25 | 1.0 |
| 6.5 | 50 | 9 | 7 | 31 | 1.3 |

**Table S3.** Thermal stability of cl-Par-4 at neutral pH by deconvolution of the CD spectra.

| Temperature (ºC) | Helix (%) | β-Sheet (%) | Turn (%) | Disorder (%) | ϴ_222_/ϴ_208_ |
| --- | --- | --- | --- | --- | --- |
| 5 | 68 | 3 | 11 | 18 | 2.6 |
| 25 | 56 | 5 | 18 | 21 | 1.9 |
| 45 | 56 | 5 | 18 | 21 | 1.6 |
| 65 | 40 | 13 | 18 | 29 | 1.0 |
| 85 | 28 | 17 | 21 | 34 | 0.6 |

**Table S4.** Thermal stability of cl-Par-4 at acidic pH by deconvolution of the CD spectra.

| Temperature (ºC) | Helix (%) | β-Sheet (%) | Turn (%) | Disorder (%) | ϴ_222_/ϴ_208_ |
| --- | --- | --- | --- | --- | --- |
| 5 | 81 | 0 | 6 | 15 | 1.4 |
| 25 | 79 | 2 | 6 | 14 | 1.3 |
| 45 | 81 | 0 | 5 | 15 | 1.2 |
| 65 | 56 | 5 | 17 | 23 | 1.1 |
| 85 | 21 | 24 | 19 | 36 | 0.7 |
